# Endorsement of a single-item measure of sleep disturbance during pregnancy and risk for postpartum depression: a retrospective cohort study

**DOI:** 10.1007/s00737-022-01287-9

**Published:** 2023-01-12

**Authors:** Jennifer N. Felder, Danielle Roubinov, Li Zhang, Mark Gray, Arne Beck

**Affiliations:** 1 Department of Psychiatry and Behavioral Sciences, University of California, San Francisco, CA USA; 2 Osher Center for Integrative Health, University of California, San Francisco, CA USA; 3 Department of Medicine, University of California, San Francisco, CA USA; 4 Department of Epidemiology and Biostatistics, University of California, San Francisco, CA USA; 5 Institute for Health Research, Kaiser Permanente Colorado, Aurora, CO USA

**Keywords:** Insomnia, Sleep quality, Sleep disturbance, Prenatal, Perinatal depression, Prevention of depression

## Abstract

Poor prenatal sleep quality is associated with increased risk for depressive symptoms but may go undetected in brief, busy prenatal care visits. Among non-depressed pregnant participants, we evaluated whether 1) the endorsement of sleep disturbance on a depression questionnaire predicted postpartum depressive symptoms, 2) the strength of these associations was higher than other somatic symptoms of pregnancy and depression (i.e., fatigue, appetite disturbance), and 3) the endorsement of prenatal sleep disturbance varied by participant characteristics. In this retrospective cohort study, participants had a live birth and completed Patient Health Questionnaire (PHQ-9) during pregnancy and within 8 weeks postpartum between 2012 and 2017. Participants who were non-depressed during pregnancy (PHQ-9 < 10) were included (*n* = 3619). We operationalized sleep disturbance, fatigue, and appetite disturbance as endorsement of item 3, 4, and 5 on the PHQ-9, respectively, and postpartum depressive symptoms as PHQ-9 total score ≥ 10. Participant characteristic variables included age, race, ethnicity, parity, gestational age at delivery, and preterm birth. Prenatal sleep disturbance was associated with higher odds of postpartum depressive symptoms (aORs 1.9, 95% CI 1.2–3.1 for first trimester; 3.7, 95% CI 1.5–11.5 for second trimester; 3.4, 95% CI 1.9–6.8 for third trimester). Fatigue and appetite disturbance in the first and third trimesters were associated with higher odds of postpartum depressive symptoms. Sleep disturbance varied by race during the first and second trimesters (*p* < 0.05) and was highest among Black or African American participants (61.8–65.1%). A routinely administered single-item measure of sleep disturbance could identify otherwise lower-risk pregnant individuals who may benefit from depression prevention efforts.

**Supplementary Information:**

The online version contains supplementary material available at 10.1007/s00737-022-01287-9.

## Introduction

Poor sleep quality is highly prevalent across all months of pregnancy (Mindell et al. 2015), with important implications for maternal health. For example, poor sleep quality is associated with increased risk of perinatal depression (Skouteris et al. 2008; Baglioni et al. 2011); suicidal ideation after adjusting for depressive symptoms (Gelaye et al. 2016); preterm birth (Blair et al. 2015; Okun et al. 2011); and caesarean birth (Li et al. 2016; Lee and Gay 2004). A growing body of research shows that it is possible to improve sleep during pregnancy. For example, three randomized controlled trials document the efficacy of cognitive behavioral therapy for insomnia among pregnant participants^1^with insomnia symptoms or insomnia disorder (Felder et al. 2020; Manber et al. 2019; Kalmbach et al. 2020). Further, treating prenatal insomnia may prevent postpartum depression (Felder et al. 2021; Khazaie et al. 2013). Although it is prudent to assess sleep during pregnancy, there are important barriers such as lack of time during prenatal care visits or the perception that sleep disturbance is a normative, harmless symptom of pregnancy (Felder et al. 2019).

Depression screening in routine prenatal care is becoming more common in response to clinical guidelines (The American College of Obstetricians and Gynecologists 2015) and recommendations (Siu et al. 2016). The Patient Health Questionnaire-9 (PHQ-9) and the Edinburgh Postnatal Depression Scale (EPDS) are frequently used in prenatal care to screen for depression, and both include an item that assesses sleep disturbance (Kroenke et al. 2001a; Cox et al. 1987). However, clinical decisions for additional follow-up, treatment, or referral are typically based upon whether the total score exceeds a pre-specified cut-off value (e.g., PHQ-9 total score ≥ 10). Given robust associations between poor sleep quality and the development of depression, a single item assessing sleep disturbance could be an efficient prognostic indicator of those who may benefit from prevention intervention, even if the total score is below the clinical cut point indicative of risk for current depression.

To this end, the primary goal of this paper was to examine whether a single item measure of sleep disturbance is a useful tool for predicting postpartum depression. Specifically, we examined whether prenatal endorsement of sleep disturbance on the nine-item PHQ-9 (Kroenke et al. 2001a)— in the absence of current depression—predicted elevated depressive symptoms at approximately 6 weeks postpartum. Second, to explore whether the sleep disturbance item was unique in its association with elevated postpartum depressive symptoms, we also examined prospective associations with endorsement of the two other PHQ-9 items that address somatic symptoms of depression that also can be conflated with normative pregnancy symptoms—fatigue and appetite disturbance. Finally, to aid in identification of those most at risk of experiencing sleep disturbance during pregnancy, and because previous research has found racial and ethnic differences in sleep duration, self-reported troubled sleeping, and elevated insomnia symptom severity (Amyx et al. 2017; Kalmbach et al. 2019), we investigated the participant characteristics of pregnant participants who endorsed the sleep disturbance item of PHQ-9.

## Methods

### Participants and setting

**Fig. 1:**
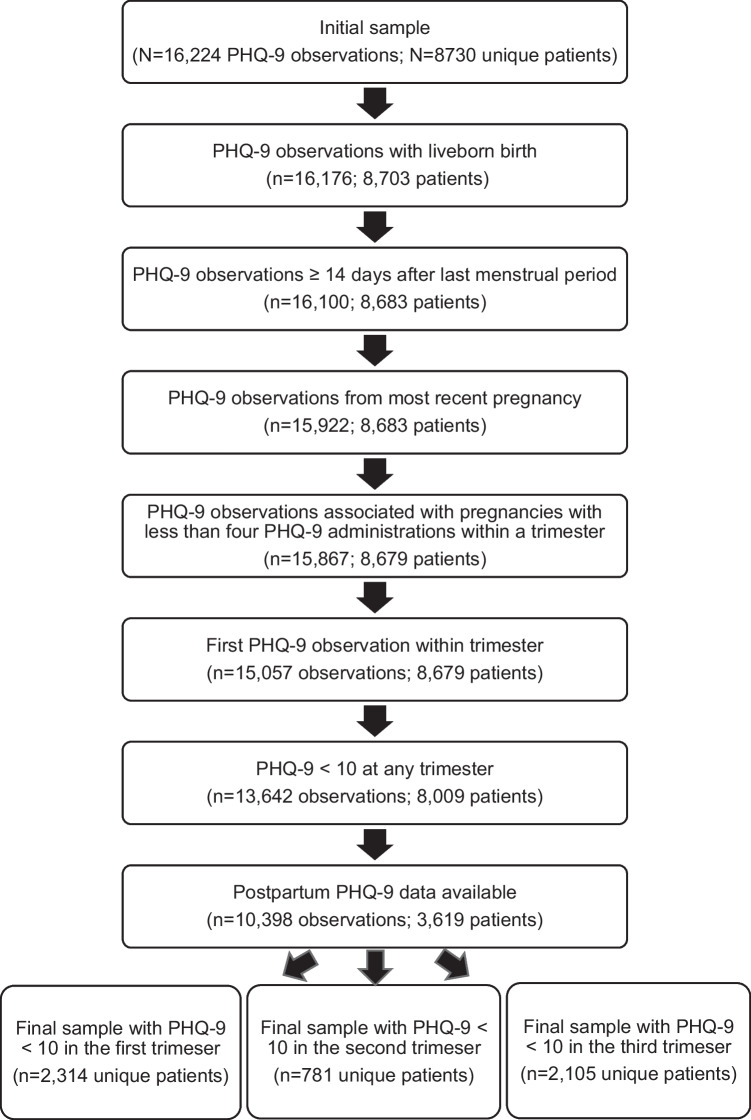
Sample selection

We conducted a retrospective cohort study in an integrated healthcare system that provides health insurance and clinical services to more than 640,000 individuals in the metropolitan Denver area and northern and southern CO communities. The study cohort included perinatal patients between 2012 and 2017 who had a live birth and who had completed PHQ-9 documented in the electronic health record between their pregnancy onset date and 8 weeks postpartum (Fig. 1). For participants with multiple pregnancies in the dataset, only the most recent was included. Outlier participants with four or more PHQ-9 administrations within a trimester were excluded. For participants with two to three PHQ-9 administrations within a trimester, the first observation was used. Finally, the analyzed sample included those with a PHQ-9 total score < 10 at any trimester, indicating minimal to mild depressive symptom severity. This study was approved by the institutional review board in the integrated healthcare system.

### Data sources and measures

All the data were extracted from the Virtual Data Warehouse (VDW), which contains electronic health record data (ICD-9/10 diagnosis codes, procedure codes, participant demographics, and participant-reported outcome data including PHQ-9) and insurance claims data.

The PHQ-9 is a nine-item self-report measure of depressive symptom severity (Kroenke et al. 2001b). The sleep disturbance item (#3) assesses both insomnia and hypersomnia (“trouble falling asleep or staying asleep, or sleeping too much”). The fatigue item (#4) assesses feeling tired or having low energy. The appetite disturbance item (#5) assesses both poor appetite and overeating. Respondents indicate how often they have been bothered by symptoms, with response options including “not at all (0),” “several days (1),” “more than half the days (2),” or “nearly every day (3).” Endorsement of these items was defined as any score above “not at all (0).” Total scores range from 0 to 27, and scores of 10 or above are typically used to identify possible depression. In the present study, non-depressed pregnant participants were defined as those with PHQ scores < 10. In this health system, PHQ-9 is administered during the first and second trimesters of pregnancy and at 5–8 weeks postpartum.

Gestational length (based on ultrasound dating) and other participant characteristics (age at the first trimester visit, Hispanic ethnicity, race, and parity) were also extracted from the VDW for statistical analyses.

### Statistical analyses

Participant characteristics were summarized by descriptive statistics. Frequency distribution and percentage were used to summarize categorical variables, and mean with standard deviation (SD) was used to summarize continuous variables.

First, to investigate prospective associations between prenatal endorsement of sleep disturbance and elevated postpartum depressive symptoms (PHQ-9 ≥ 10), univariate and multivariate logistic regression models were used, with separate models for each pregnancy trimester. Covariates with documented associations with postpartum depression were selected for inclusion in the models (Guintivano et al. 2018; Hassdenteufel et al. 2021). The same analytic approach was used for investigating prospective associations between prenatal endorsement of fatigue and elevated postpartum depressive symptoms, and prospective associations between prenatal endorsement of appetite disturbance and elevated postpartum depressive symptoms. Because the predictor variable (i.e., sleep disturbance, fatigue, or appetite disturbance) was included in the outcome variable (PHQ-9 total score ≥ 10) albeit at different timepoints, we conducted sensitivity analyses that omitted this variable from the outcome variable and used a PHQ-9 cutoff that was pro-rated for eight items (i.e., PHQ-9 ≥ 8.89).

Second, to aid in the identification of those most at risk of experiencing sleep disturbance during pregnancy, we examined participant characteristics that correlated with endorsement of sleep disturbance. Specifically, two-sample *t*-tests were used for continuous variables and chi-squared test for categorical variables. Statistical significance was declared at *p* < 0.05, and all the statistical analyses were done by the statistical computing software R.

## Results

### Participants

A total of 3619 participants were eligible for analysis (see Fig. 1 for sample selection details). On average, the participants were 31 years old, and the majority was White, non-Hispanic, and multiparous (see Table 1).

**Table 1:** Participant characteristics for the analyzed sample

Participant characteristics (*n* = 3619)	M (SD) or *n* (%)
Age at first trimester (years)	31.54 (5.07)
Race	
White	2487 (68.7%)
Unknown	565 (15.6%)
Asian	234 (6.5%)
Black or African American	174 (4.8%)
Other, native Hawaiian or other Pacific Islander, American Indian/Alaska native	159 (4.4%)
Ethnicity	
Non-Hispanic	2850 (78.8%)
Hispanic	723 (20.0%)
Unknown	46 (1.3%)
Parity	
Multiparous	1932 (53.4%)
Nulliparous	1681 (46.4%)
Unknown	6 (0.2%)
Gestational age at delivery (weeks)	39.08 (1.93)
Preterm birth	276 (7.6%)

### Prediction of postpartum depression (Table 2)

First, we investigated the prospective associations between prenatal endorsement of sleep disturbance and elevated postpartum depressive symptoms. Among pregnant participants who were not depressed (i.e., PHQ-9 total < 10), endorsement of sleep disturbance at any trimester was associated with increased risk of elevated postpartum depression symptoms in adjusted analyses. Endorsement of sleep disturbance in the second and third trimesters was associated with over three-fold higher odds of elevated postpartum depressive symptoms, adjusting for age, race, ethnicity, and parity (second trimester sleep disturbance aOR 3.74, 95% CI 1.47–11.49, *p* = 0.01; third trimester sleep disturbance aOR 3.43, 95% CI 1.88–6.78, *p* < 0.001), followed by nearly twofold higher odds for endorsement of sleep disturbance in the first trimester (aOR 1.90, 95% CI 1.17–3.13, *p* = 0.009).

**Table 2:** Univariate and multivariate associations between sleep disturbance, fatigue, and appetite disturbance by trimester and elevated postpartum depressive symptoms

	First trimester *n* = 2314		Second trimester *n* = 781		Third trimester *n* = 2104	
	Univariate OR (95% CI, *p*-value)	Multivariate OR (95% CI, *p*-value)	Univariate OR (95% CI, *p*-value)	Multivariate OR (95% CI, *p*-value)	Univariate OR (95% CI, *p* value)	Multivariate OR (95% CI, *p*-value)
Sleep disturbance	**1.71 (1.11–2.66,** ***p*** **= 0.016)**	**1.90 (1.18–3.13,** ***p*** **= 0.009)**	**4.66 (1.93–13.87,** ***p*** **= 0.002)**	**3.74 (1.47–11.49,** ***p*** **= 0.010)**	**2.67 (1.63–4.60,** ***p*** **< 0.001)**	**3.43 (1.88–6.78,** ***p*** **< 0.001)**
Fatigue	**3.21 (1.33–10.56,** ***p*** **= 0.023)**	**3.44 (1.27–14.11,** ***p*** **= 0.038)**	1.97 (0.85**–**5.35, *p* = 0.141)	1.42 (0.58**–**3.99, *p* = 0.463)	**2.19 (1.25–4.16,** ***p*** **= 0.010)**	**2.24 (1.18–4.71,** ***p*** **= 0.021)**
Appetite disturbance	**1.91 (1.24–3.00,** ***p*** **= 0.004)**	**2.10 (1.30–3.47,** ***p*** **= 0.003)**	**2.32 (1.06–4.83,** ***p*** **= 0.028)**	2.01 (0.78**–**4.76, *p* = 0.124)	**2.27 (1.41–3.56,** ***p*** **= 0.001)**	**2.15 (1.23–3.65,** ***p*** **= 0.006)**

Bolded cells indicate *p*-values < 0.05. Multivariable models adjusted for age, race, ethnicity, and nulliparity

Secondary analyses examined the prospective association of other somatic symptoms of pregnancy with postpartum depression. In the first and third trimesters, both fatigue and appetite disturbance were associated with increased odds of elevated postpartum depressive symptoms in adjusted analyses. For example, endorsement of fatigue in the first trimester was associated with over three-fold higher odds of elevated postpartum depressive symptoms in the adjusted analyses (OR 3.44, 95% CI 1.27–14.11, *p* = 0.038). Endorsement of appetite disturbance in the third trimester was associated with over two-fold higher odds of elevated postpartum depression symptoms in the adjusted analyses (OR 2.15, 95% CI 1.23–3.65, *p* = 0.006).

Third trimester sleep disturbance had a stronger association with elevated postpartum depressive symptoms (aOR 3.43) than did the other somatic symptoms (fatigue aOR 2.24; appetite disturbance aOR 2.15). Additionally, second trimester sleep disturbance, but not fatigue or appetite disturbance, was associated with increased risk of elevated postpartum depression symptoms in the adjusted analyses (aOR 3.74, 95% CI 1.47–11.49, *p* = 0.01).

The results did not substantively change in sensitivity analyses, suggesting that the associations between prenatal sleep disturbance and elevated postpartum depressive symptoms were not driven by the association between prenatal sleep disturbance and postpartum sleep disturbance. The same was true for the adjusted analyses of appetite disturbance. In contrast, in the adjusted analyses, fatigue no longer had a statistically significant association with elevated postpartum depressive symptoms when omitting the fatigue item (see Supplemental Table 1).

### Correlates of prenatal sleep disturbance (Table 3)

Endorsement of sleep disturbance varied significantly by race during the first and second trimesters but not the third trimester. For example, 48.4% of Asian participants reported sleep disturbance in the first trimester compared to 65.1% of Black or African American participants. During the first two trimesters, endorsement of sleep disturbance was highest among the Black or African American participants (ranging from 61.8 to 65.1%) and lowest among the Asian participants (ranging from 33.3 to 56.2%). Endorsement of sleep disturbance also varied significantly by parity during the first and third trimesters, such that sleep disturbance was less common among nulliparous participants.

**Table 3:** Correlates of sleep disturbance by timepoint (M (SD) for continuous variables and *n* (%) for categorical variables)

	First trimester			Second trimester			Third trimester		
	No sleep disturbance	Sleep disturbance	*p-*value	No sleep disturbance	Sleep disturbance	*p-*value	No sleep disturbance	Sleep disturbance	*p-*value
*n*	1636	1759		523	662		1207	1673	
Age at first trimester (years)	32.05 (4.86)	31.23 (5.34)	< 0.001	31.31 (5.27)	31.15 (5.75)	0.625	31.55 (5.04)	31.11 (5.18)	0.022
Race			< 0.001			0.001			0.685
White	1182 (50.0)	1181 (50.0)		336 (43.0)	445 (57.0)		832 (42.5)	1126 (57.5)	
Unknown	227 (45.8)	269 (54.2)		80 (41.2)	114 (58.8)		186 (39.7)	283 (60.3)	
Asian	99 (51.6)	93 (48.4)		54 (66.7)	27 (33.3)		81 (43.8)	104 (56.2)	
Black or African American	65 (34.9)	121 (65.1)		28 (38.4)	45 (61.6)		50 (38.2)	81 (61.8)	
Other, native Hawaiian or other Pacific Islander, American Indian/Alaska native	63 (39.9)	95 (60.1)		25 (44.6)	31 (55.4)		58 (42.3)	79 (57.7)	
Ethnicity			0.147			0.481			0.697
Non-Hispanic	1333 (49.0)	1386 (51.0)		413 (45.0)	505 (55.0)		945 (42.1)	1302 (57.9)	
Hispanic	283 (44.8)	349 (55.2)		104 (41.6)	146 (58.4)		249 (41.8)	347 (58.2)	
Unknown	20 (45.5)	24 (54.5)		6 (35.3)	11 (64.7)		13 (35.1)	24 (64.9)	
Parity			< 0.001			0.163			0.008
Multiparous	658 (43.9)	841 (56.1)		219 (41.2)	313 (58.8)		513 (38.9)	807 (61.1)	
Nulliparous	965 (51.7)	901 (48.3)		300 (46.7)	343 (53.3)		691 (44.6)	860 (55.4)	
Unknown	13 (43.3)	17 (56.7)		4 (40.0)	6 (60.0)		3 (33.3)	6 (66.7)	
Gestational age at delivery (weeks)	273.42 (14.21)	272.72 (13.67)	0.144	274.59 (11.83)	273.35 (12.53)	0.083	274.74 (11.64)	274.46 (11.21)	0.523
Preterm birth	136 (44.3)	171 (55.7)	0.171	35 (39.3)	54 (60.7)	0.401	87 (43.3)	114 (56.7)	0.737

## Comment

### Principal findings

Our findings show that pregnant participants without clinically significant depression symptoms who reported “trouble falling or staying asleep or sleeping too much” on the  PHQ-9 at any trimester were at increased risk for elevated depressive symptoms at 6 weeks postpartum. Endorsement of fatigue and appetite disturbance, which also are somatic symptoms of both pregnancy and depression, was associated with increased risk of elevated postpartum depressive symptoms. However, endorsement of sleep disturbance in the second trimester was unique in predicting postpartum depression.

### Results in the context of what is known

These findings are consistent with previous research reporting similar associations using longer, multiple-item assessments of prenatal insomnia (Tomfohr et al. 2015). For example, non-depressed pregnant participants who reported nightly difficulties falling asleep during the third trimester were nearly four times as likely to experience depression by 6 months postpartum compared to those who reported no or occasional difficulty falling asleep (24% vs 7%, respectively) (Suri et al. 2017). Findings from the current paper add to the evidence base by showing that even a single-item measure of sleep disturbance has utility in prospectively identifying risk for postpartum depression.

Our findings are also consistent with previous research showing that pregnant women who are Black are more likely to report short or very short sleep (≤ 6 h per night) relative to those who are non-Hispanic White (Amyx et al. 2017). Racial disparities in endorsement of sleep disturbance are likely a proxy for other unmeasured factors; for example, research among perinatal samples pinpoints racism and discrimination as a driver of poor sleep in this population (Gaston et al. 2020; Slopen et al. 2016). This finding is particularly notable in the context of disparities in access to and/or quality of perinatal care that may exacerbate the burden of prenatal sleep disturbance among Black or African American people (Vedam et al. 2019; McLemore et al. 2018).

### Clinical implications

Our findings suggest that item 3 of the PHQ-9 may be an efficient tool for identifying a risk factor for postpartum depression that may otherwise be under-recognized or even dismissed as a normative, harmless symptom of pregnancy (Felder et al. 2019). Clinically, this suggests that providers could examine the endorsement of sleep disturbance item on PHQ-9 to identify risk for *future* depression, in addition to examining total scores to identify *current* depression. This is akin to standard clinical practice that necessitates following up on endorsement of the suicidal ideation item of PHQ-9 even when the PHQ-9 total score is below the conventional cutoff of 10.

Our findings suggest that clinicians should be particularly attentive to endorsement of sleep disturbance in the second trimester as it may be a “canary in the coal mine” that is unique relative to other somatic symptoms of pregnancy in portending risk for depression. Additionally, unlike fatigue and appetite disturbance, sleep disturbances, particularly insomnia, have evidence-based treatments. A randomized controlled trial found that 64% of pregnant participants who received in-person cognitive behavioral therapy for insomnia (CBT-I) experienced symptom remission at post-intervention (Manber et al. 2019). There is also evidence that digital adaptations of CBT-I are effective during pregnancy (Felder et al. 2020; Kalmbach et al. 2020). Of particular relevance is the increasing evidence that CBT-I may prevent depression among adults with insomnia, older adults, and perinatal people (Felder et al. 2021; Cheng et al. 2019; Irwin et al. 2022). If left untreated, prenatal sleep disturbance can persist into the postpartum period (Tomfohr et al. 2015). The US Preventive Services Task Force recommends that pregnant people at risk for depression receive preventive counseling interventions (U. S. Preventive Services Task Force et al. 2019). Taken together, these findings underscore the importance of identifying and treating prenatal sleep disturbances.

### Limitations

The results of the present study should be considered in the context of several limitations. First, the single PHQ-9 sleep disturbance item assesses experiences of insomnia and hypersomnia; thus, we are unable to examine whether there are differences in the degree of risk conferred between these indicators. Second, data on depressive symptoms was only available through 6 weeks postpartum. The onset and course of postpartum depressive symptoms are highly variable and may emerge or persist up to 12 months following childbirth (American Psychiatric Association 2013). Although we were unable to investigate whether endorsement of sleep disturbance at 6 weeks postpartum predicted risk for elevated depressive symptoms later in the postpartum period, previous research suggests that mothers who reported having problems with sleep at 6 weeks postpartum were more likely to have elevated depressive symptoms at 6 or 12 weeks postpartum (Stremler et al. 2020). Relatedly, the focus of the current study was on elevated depressive symptoms (i.e., PHQ-9 > 10); although scores in this range are suggestive of depression, it is possible that the present results may not generalize to those with clinical diagnoses of depression. It is notable, however, that even subclinical depressive symptoms exert a substantial impact on maternal and child functioning (Meaney 2018). Third, although the present study is strengthened by its inclusion of a large sample of individuals drawn from a managed healthcare organization, the racial and ethnic background is not representative of the birthing population in the USA. For example, the Center for Disease Control 2018 Natality Data indicate that 52% of births were to a non-Hispanic White mother compared to the 69% in the current sample. Fourth, we were unable to examine whether prenatal sleep disturbance varied across other potentially important clinical characteristics such as history of depression or family mental health history. Finally, we acknowledge that the risk for depressive symptoms conferred by sleep disturbance was examined using a sleep item on a measure that also contributed to the total depressive symptom score derived from that same measure (i.e., item 3 on PHQ-9 assesses sleep disturbance and is also part of the total summed score on PHQ-9). However, this approach may align with the “real-world” conditions of busy obstetrics practices that may not have the time or providers available to administer and score separate measures of depression and sleep disturbance.

## Conclusions

Sleep disturbance is frequently experienced by pregnant individuals and may operate as a robust risk factor for a number of adverse postpartum health outcomes. Although measures of sleep are not frequently administered as part of prenatal care, singular sleep items are often embedded within routine depression screenings. The present study found that among non-depressed pregnant participants, sleep disturbance, as assessed by a single item from PHQ-9, was associated with significantly increased risk of elevated postpartum depression symptoms. Endorsement of sleep disturbance in the second trimester may be an early predictor of postpartum depression. Notably, the risk for later depression conferred by prenatal sleep disturbance was greater than that of other somatic symptoms that similarly may be deemed normative during pregnancy (i.e., fatigue, appetite disturbance). We also observed that sleep disturbance was disproportionately endorsed by Black or African American participants relative to participants of other racial backgrounds, potentially highlighting those for whom monitoring and addressing sleep disturbances during pregnancy is particularly important. Taken together, our results suggest that a brief, easily administered, stand-alone measure of sleep disturbance could help identify otherwise lower-risk individuals who may benefit from prevention efforts during pregnancy.

## Supplementary Information

Below is the link to the electronic supplementary material.

Supplementary file1 (DOCX 19 KB)

## Footnotes

1 Because our dataset did not have information about gender identity, we use the term “participants” instead of “women,” with the caution that more research is needed to examine the extent to which current findings depend on gender identity.
