# Supplementary material for: Endorsement of a single-item measure of sleep disturbance during pregnancy and risk for postpartum depression: a retrospective cohort study
Source: Arch Womens Ment Health. 2023 Jan 12;26(1):67–74. doi: 10.1007/s00737-022-01287-9 (PMC9908713; doi:10.1007/s00737-022-01287-9)
Supplement: Supplementary file 1 — Supplementary file1 (DOCX 19 KB) [file 737_2022_1287_MOESM1_ESM.docx]

**Supplemental Table 1**. Sensitivity analyses displaying univariate and multivariate associations between sleep disturbance, fatigue, and appetite disturbance by trimester and elevated postpartum depressive symptoms (PHQ-9 ≥ 8.89).

|  | First trimester  n=3395 | | Second trimester  n=1185 | | Third trimester  n=2880 | |
| --- | --- | --- | --- | --- | --- | --- |
|  | Univariate OR (95% CI, p value) | Multivariate OR (95% CI, p value) | Univariate OR (95% CI, p value) | Multivariate OR (95% CI, p value) | Univariate OR (95% CI, p value) | Multivariate OR (95% CI, p value) |
| Sleep disturbance | **1.78 (1.13-2.84, p=0.014)** | **2.03 (1.24-3.43, p=0.006)** | **7.20 (2.50-30.42, p=0.001)** | **6.25 (2.10-26.86, p=0.004)** | **2.16 (1.28-3.82, p=0.005)** | **2.78 (1.50-5.56, p=0.002)** |
| Fatigue | **3.29 (1.21-13.49, p=0.045)** | 3.90 (1.21-23.94, p=0.060) | 2.26 (0.85-7.85, p=0.139) | 1.80 (0.64-6.38, p=0.305) | 1.90 (1.02-3.85, p=0.056) | 2.06 (1.01-4.78, p=0.065) |
| Appetite disturbance | 1.47 (0.94-2.33, p=0.095) | **1.64 (1.02-2.71, p=0.046)** | **2.21 (1.01-4.57, p=0.037)** | 1.65 (0.65-3.81, p=0.263) | **2.08 (1.26-3.36, p=0.003)** | **2.28 (1.28-3.92, p=0.004)** |

*Note****.*** Bolded cells indicate p values < .05. Multivariable models adjust for age, race, ethnicity, and nulliparity.
